# A new species of *Oxelytrum* Gistel (Coleoptera, Silphidae) from southern Argentina, with a key to the species of the genus

**DOI:** 10.3897/zookeys.203.2837

**Published:** 2012-06-20

**Authors:** Adriana Oliva

**Affiliations:** 1 Laboratorio de Entomología forense, Museo argentino de Ciencias naturales, Avenida Ángel Gallardo 470, C1405DJR, Buenos Aires, Argentina

**Keywords:** Silphidae, forensic entomology, Neotropical fauna, Patagonian fauna

## Abstract

A new species of the forensically interesting genus *Oxelytrum* Gistel (Coleoptera, Silphidae), *Oxelytrum selknan*, is described from Santa Cruz and Tierra del Fuego provinces, Argentina. The new species resembles *Oxelytrum biguttatum* (Philippi) in outer aspect, but has different male genitalia, in particular a median lobe longer than the paramera. All the described species of *Oxelytrum* have the median lobe shorter than the paramera. The internal sac, as far as it could be reconstructed from dry-pinned specimens, also shows differences between the two species. A key to the species of *Oxelytrum* is given and illustrated.

## Abstract (es)

Se describe una nueva especie de *Oxelytrum* Gistel (Coleoptera, Silphidae), género de interés forense: *Oxelytrum selknan*, de las provincias de Santa Cruz y Tierra del Fuego, Argentina. La nueva especie se parece en aspecto exterior a *Oxelytrum biguttatum* (Philippi), pero tiene genitales masculinos diferentes, en particular el lóbulo mediano más largo que los parámeros. Todas las especies de *Oxelytrum* descriptas tienen el lóbulo mediano más corto que los parámeros. El saco interno, en la medida en que se pudo reconstituir a partir de especímenes secos y montados, muestra también diferencias entre las dos especies. Se presenta una clave para las especies de *Oxelytrum*, con ilustraciones.

## Introduction

The family Silphidae, in its present sense, comprises 25 genera with some 200 species distributed all over the world (Newton and Thayer in Pakaluk and Slipinsky 1995). The adults of this family are rather large beetles, without ocelli, with elytra punctate but never striate, and with 6–7 differentiated ventrites (Hansen 1997). The male genitalia are of the trilobed type, with an internal sac (Blackburn 1930). The species of Silphidae are necrophages or predators, sometimes a combination of both behaviors. Adults of corpse-frequenting species prey on the eggs and larvae of flies (Dorsey 1940; Payne and King 1970; Oliva and Di Iorio 2008). In the subfamily Nicrophorinae Kirby there is parental care of larvae, which has not been observed in the subfamily Silphinae Latreille. The larvae have maxilae with large, wide mala, divided in the apical ¼; galea with a brush of dense hairs, articulate urogomphi, which may be uni- or bisegmented (Hansen 1997).

The species of Silphidae occurring in Latin America were revised by Peck and Anderson (1985), who mentioned that no larvae had been described for South America. Later, the larvae of *Oxelytrum erythrurum* (Oliva, 2005) and of *Oxelytrum discicolle* (Velásquez and Viloria 2009, 2010) were studied. Oliva and Di Iorio (2008) reviewed the species found in Argentina and corrected some of the locality names given by Peck & Anderson. The latter treated all of Latin America, and thus mention the genera *Necrodes*, *Heterosilpha*, *Oiceoptoma* and *Thanatophilus* besides *Nicrophoru*s and *Oxelytrum* which are the only genera found in South America. A paper on the Ibero-American Silphidae in the collection of the Museo Nacional de Ciencias Naturales of Madrid, Spain, treats five species of the genus *Oxelytrum* and one of the genus *Nicrophorus* (Pérez Valcárcel et al. 2012).

### The genus *Oxelytrum* Gistel

**Figure 1.**
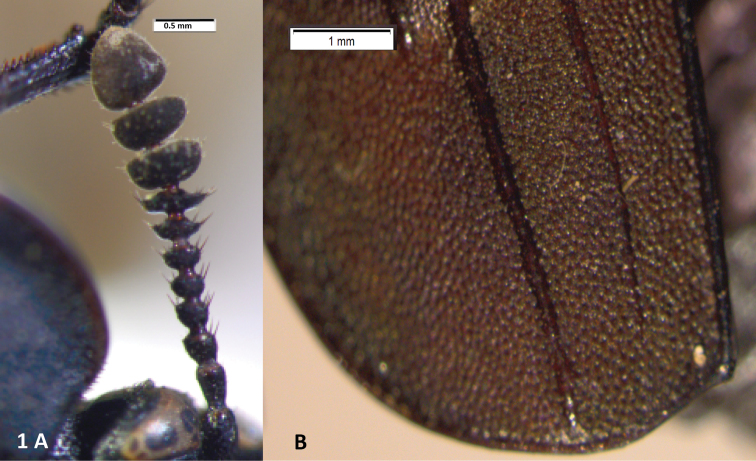
*Oxelytrum selknan* sp. n. **A** antenna, showing antennomere 8 dilated into a disk. Scale bar= 0.5 mm. **B** elytral apex (male), showing gently curved outer margin and posterior margin weakly produced near suture. Scale bar= 1 mm.

A Neotropical genus: only *Oxelytrum discicolle* reaches the S of USA (Peck and Andersen 1985). Flat, rather large beetles (12–20 mm in length). Antennae (Fig. 1A) gently broadened towards the apex into a three-segmented club, preceded by an antennomere modified in shape, a little like the cupula of Hydrophiloidea (Newton and Thayer 1992; Hansen 1992). Protarsi of the males slightly dilated at base, with thick, stiff hairs on the contact surface (Fig. 10A, 10B). Hairs strongly curved at the apex (Fig. 9C), not dilated. Pronotum wide, laterally expanded; disk, more or less strongly raised according to species, with longitudinal costae that may be obsolete (Fig. 8A). Elytra densely punctate, bearing raised costae (Figs 5, 8A). Humeral humps of elytra projecting, rounded (Fig. 5) or toothed (Fig. 8B). Elytral apices produced (Fig. 8A) or not (Fig. 5). General color black or very dark brown, according to the age of the specimen. Specific color patterns are described in the key. Male genitalia trilobed, fairly well sclerotized; they provide good diagnostic characters.

### Silphidae: male genitalia

Partly because female genitalia give better diagnostic characters in other families of Staphylinoidea, partly because this genus has external species-diagnostic characters, very little attention has been given to the male genitalia of *Oxelytrum*. Blackburn (1936) and Háva et al. (1999) have described male genitalia in other genera of Silphidae.

The purpose of the present work was to describe the male genitalia of the species of *Oxelytrum* found in Argentina. It was found that what seemed one widely distributed species was comprised of two, one of them new to science.

Male genitalia of Silphidae Silphinae are trilobed, with a membranous internal sac; the median lobe is well sclerotized on the ventral surface and membranous on the dorsal surface; the ejaculatory duct enters the median lobe ventral to the basal piece; there is an internal sac (Blackburn, 1930). In the species of *Oxelytrum*, the basal piece proved small and weakly sclerotized, so it was not taken into account. The paramera are always narrow,. The median lobe is straight and sturdy in all the species examined, with a complicated internal sac, which sometimes can be extruded after relaxing and clearing.

### Silphidae and forensic entomology

Forensic entomology studies the insects found on a corpse, with the purpose of dating the death. Sometimes, circumstances that surrounded or followed the death can be discovered, but not always.

Silphidae of the genus *Oxelytrum* develop on large animal carcasses. Of six species found in Argentina, four have been found in autopsies (Oliva and Di Iorio 2008). One of these species, *Oxelytrum discicolle*, is tropical and subtropical in distribution and has been found in other South American countries (Velásquez and Viloria 2009). Larvae of *Oxelytrum* are found under the carcass, at least during the day. It is noticeable that the soil beneath is usually hardened by seeping of cadaveric fluids. Oliva (2004) suggested that the larvae may feed on these fluids, and that this is the reason for the absence of a mola in their mandibles. Adult *Oxelytrum erythrurum* may appear very early in the succession, behaving as necrophiles (predators on necrophages), small larvae together with the adults may occur around 10–15 days from death, and after 20–25 days, large larvae are found, usually without adults. These intervals are for Buenos Aires and Buenos Aires province (Oliva and Di Iorio 2008).

The genus *Nicrophorus* Fabricius is noted for exploiting small carcasses, which the adults bury. Adults may occasionally be found near large carcasses, but there is no evidence that they feed on them. No species of *Nicrophorus* has been found so far on human corpses.

## Material and methods

The material examined is in the collections of the Museo argentino de Ciencias naturales “Bernardino Rivadavia” (MACN), Buenos Aires, Argentina. Apart from a few lots collected in recent years and preserved in ethanol, the specimens were dry-mounted. For this study they were relaxed in boiling water and dissected. The extracted pieces were put into plastic genitalia vials with a drop of glycerine, and the pin was run through the vial plug. The male genitalia were cleared in cold NaOH for two to four days; this sometimes permitted to study the internal sac. Additional material from the Museo Nacional de Historia Natural (MNHN), Santiago, Chile, was examined. All the specimens were dry-mounted.

Photographs of male genitalia and of external morphological characters mentioned in the key were taken with an Olympus DPL5 camera adapted in an Olympus SZX16 stereomicroscope.

Most of the pinned specimens were collected in the first half of the twentieth century. The localities are often indicated vaguely, sometimes limited toa province name.

## Results

### Description of new species

#### Oxelytrum selknan sp. n.

urn:lsid:zoobank.org:act:A2420D45-607E-4EFF-B220-853A3C364E02

http://species-id.net/wiki/Oxelytrum_selknan

Oxelytrum biguttatum : Oliva & Di Iorio, 2008. Tierra del Fuego, forensic sample.

##### Description.

Large (13–16,5 mm in length), sturdy beetles, black in color, with a pair of subquadrate reddish spots on the margin of the pronotum. Antennomere 8 expanded into a disk (Fig. 1A). Eyes small, not prominent (Fig. 1A). Dorsum of pronotum and of elytra densely punctate. Pronotum flat, bearing two pairs of indistinct longitudinal costae, the outer pair basal, short, the medial pair about two thirds of the pronotal length, not reaching the anterior or the posterior margins. Elytra bearing each three longitudinal costae, the two inner ones reaching the posterior margin, the outer one interrupted about two thirds of elytral length. Elytral apices broadly rounded, in males a little produced at the sutural angle or at the segment of the margin comprised between the two innes elytral costae (Fig. 1B); in females expanded outwards. Protarsi of males with the four basal tarsomeres dilated, bearing thick adhesive hairs forming soles. Female styli short, wide, truncate at apex (Fig. 6A). Outer appearance entirely similar to *Oxelytrum biguttatum* (Philippi) (Fig. 5).

**Figure 2.**
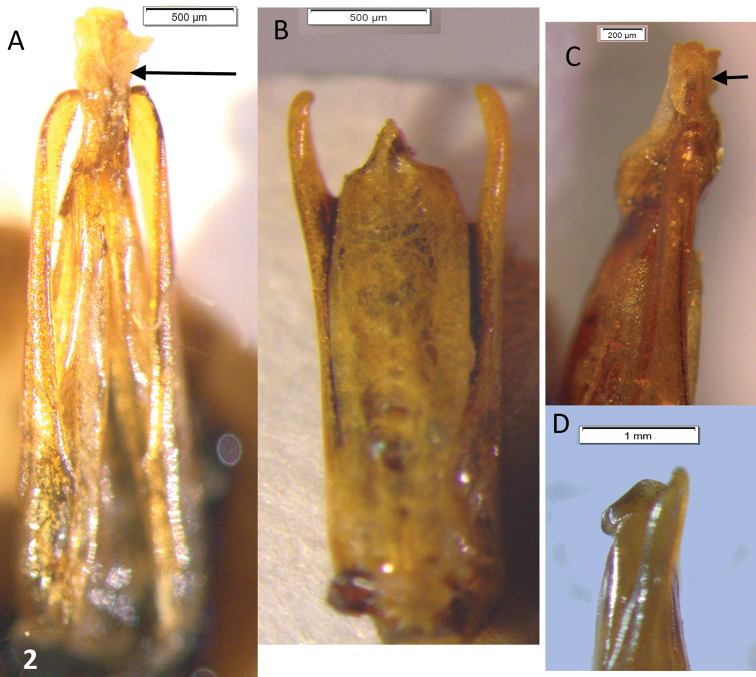
*Oxelytrum selknan* and *Oxelytrum biguttatum*, male genitalia, dry mounted. **A** *Oxelytrum selknan*, male genitalia in dorsal aspect showing median lobe longer than paramera, gradually acuminate to apex. Scale bar= 0.5 mm. **B** *Oxelytrum biguttatum*, male genitalia in dorsal aspect, showing median lobe shorter than paramera, parallel-sided, abruptly acuminate at apex. Scale bar= 0.5 mm. **C** *Oxelytrum selknan*, apex of median lobe in lateral aspect, showing acuminate apex (arrow) and rounded ventral ridge. Scale bar= 0.2 mm. **D** *Oxelytrum biguttatum*, apex of paramera and median lobe in lateral aspect, showing median lobe shorter than paramera, with rounded ventral ridge. Scale bar= 1 mm.

Male genitalia. Paramera narrow, weakly broadened in apical ⅓, apices blunt, turned inwards. Median lobe distinctly longer than paramera, in dry material triangular, narrowing evenly to the acuminate apex (Fig. 2A). Rehydrated material shows a spindle-shaped median lobe (Fig. 4A, 4B). Everted internal sac with apical cylinder covered in brown microtrichia, slightly constricted about the middle of its length; small rounded lobes basal to apical cylinder, large semi-globular lobe basal to smaller ones (Fig. 4A, 4B, 4C, 4D; small lobes marked by arrows).

##### Material examined.

Holotype, male. “Argentina/Tierra del Fuego”. 14,5 mm. Two male paratypes: Male. “Valle Túnel/Dr. Witte/ Hyponecrodes/biguttatus Phil.” 15,5 mm. Male. “Tierra del Fuego/Ushuaia/XII 1967” Leg. A. O. Bachmann. Four female paratypes: Female. “República Argentina/ Gob. Tierra del Fuego/190-/C. Bruch.” 17 mm. Female. “Tierra del Fuego” 13 mm. Female. “Argentina/Tierra del Fuego” “32938” 16,5 mm. Female. “República Argentina/Santa Cruz/ II-190-/C: Bruch.” “20959” “Hyponecrodes biguttatus”. All in the collection of the MACN.

##### Etymology.

The name alludes to the Native American people, Selk’nan (also called Ona), who inhabited the land part of the Isla Grande de Tierra del Fuego until the end of the nineteenth century.

This species can be recognized by the male genitalia. This is the only species of *Oxelytrum* known so far that has the median lobe longer than the paramera. The general shape of the internal sac is similar in *Oxelytrum selknam* sp. n. and in *Oxelytrum biguttatum* Philippi, but in the latter the apical cylinder is not constricted and bears no microtrichia apparent under 25×, there are no smaller lobes at the base of the cylinder and there are two large basal semi-globular lobes, one ventral and the other dorsal (Fig. 4E, 4F). The paramera of *Oxelytrum biguttatum* are not broadened at the apex, and the median lobe is distinctly shorter than the paramera, parallel-sided (Fig. 2AB, 2D; 3B).

**Figure 3.**
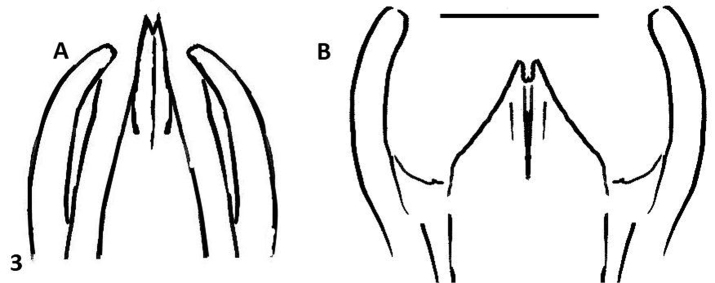
*Oxelytrum selknan* and *Oxelytrum biguttatum*, male genitalia: line drawings showing median lobe longer than paramera in the first species **A** and shorter in the other **B** Scale bar= 0.5 mm.

**Figure 4.**
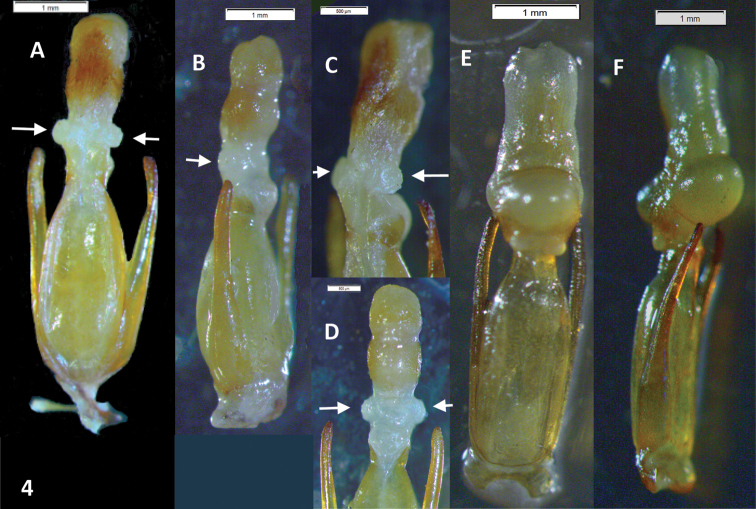
*Oxelytrum selknan* and *Oxelytrum biguttatum*, male genitalia with extruded internal sac. **A** *Oxelytrum selknan*, male genitalia in dorsal aspect, showing sac with brown microtrichia on apical portion and small rounded lobes proximal to the latter (arrows). Scale bar= 1 mm. **B** *Idem*, lateral aspect. Scale bar= 1 mm. **C** *Idem*, detail of internal sac, showing small lobes (arrows) and large basal lobe partially extruded. Scale bar= 1 mm. **D** *Idem*, ventral aspect, showing lighter coloring of apical portion and extruded small lobes (arrows). Scale bar= 0.5 mm. **E** *Oxelytrum biguttatum*, male genitalia in dorsal aspect, showing apical portion without apparent microtrichia, absence of small median lobes, and large basal lobes fully extruded. Scale bar= 1 mm. **F** *Idem*, lateral aspect. Scale bar= 1 mm

No other differences were found between *Oxelytrum selknan* sp. n. and *Oxelytrum biguttatum*. The latter species was described from Valdivia in S Chile. I have examined material from several localities in Magallanes and they all have a parallel-sided median lobe shorter than the paramera. The geographical barrier between the species appears to be the Magallanes strait. *Oxelytrum biguttatum* is found in S Chile, in the Patagonian tableland in Argentina, and in some mountain localities in SW Argentina. This species may have spread across the Andes through the present-day province of Neuquén. On the the other hand, *Oxelytrum selknan* sp. n. could have evolved in the main island of Tierra del Fuego (Isla Grande). Although the distances may appear small in the map, the straits are actually an effective barrier for flying insects. The presence of some specimens in the mainland could be explained by recent man-aided dispersion.

#### Oxelytrum biguttatum

(Philippi)

http://species-id.net/wiki/Oxelytrum_biguttatum

##### Localities.

MACN: Argentina: Tucumán: Depto Tafí Viejo: San José, 2500 m, III-1933 (26°7167'S, 65°6167'W). La Rioja: Lamadrid: Nevado de Famatina: Los Corrales: 2300–3000 m. 5-14-I-1931 (29°S,67°85'W). Río Negro (no locality). 1981, Coll. C. Bruch. MNHN: Magallanes: Ojo Bueno, 20-1-1975, Cerda. (Mainland. 53°017'S, 70°867'W). Magallanes: Parque nacional Torres del Paine, Laguna Pehué. 12-12-1986. Leg.F. Soto. (Mainland.) Two syntypes, male and female, of which only the male has a handwritten label “Valdivia”. Dr M. Elgueta (pers. com.) suggests that this may be a mistake as all the material in the collections is from Magallanes Region.

### Key to the species of *Oxelytrum* in South America

(Modified from Peck and Anderson 1985)

**Table:** 

1	Eyes small, not prominent (Fig. 5), separated by a distance clearly greater than 3× eye diameter	2
–	Eyes large, prominent (Fig. 8A), separated by a distance equivalent to 3 × eye diameter, or less	4
2	Pronotum with a small reddish spot on each of the posterolateral angles (Fig. 5)	3
–	Pronotum entirely black (dark brown in individuals that have not darkened completely)	4
3	Paramera narrow, weakly curved inwards, apices narrowly rounded (Fig. 3B). Median lobe shorter than paramera, parallel-sided, apex abruptly acuminate, with a high ventral ridge (Fig. 2D). Internal sac with apical portion subcylindrical, whitish, not bearing brown microtrichia, proximal to this portion two large hemispherical lobes (Fig. 4E, 4F)	*Oxelytrum biguttatum* (Philippi) NW Argentina above 2,300 MASL; Río Negro, Neuquén. Chile: Antártica, Magallanes; Última esperanza; Valdivia (?)(MNHN); also Aisen, Chiloé; Llanquihue; Osorno; (Peck and Anderson 1985)
–	Paramera narrow, almost straight, weakly broadened at apical ¼, apices rounded (Fig. 2A). Median lobe longer than paramera, gradually narrowed to the apex (Fig. 2A, 2C, 3A). Inner sack with cylindrical apical portion covered in brown microtrichia (Fig. 4A–4D), proximal to this three small rounded lobes, proximal to them hemispheric lobe	*Oxelytrum selknan* sp. n. Santa Cruz, Tierra del Fuego.
4	Antennal club with the three apical segments yellow or orange. Paramera narrow, straight, apices rounded and strongly turned inwards. Median lobe slightly shorter than paramera, spindle-shaped, gradually acuminate (Fig. 7A); apex narrowly rounded, with ventral ridge weakly angular in lateral aspect (Fig. 7B, 7C)	*Oxelytrum apicale* (Brullé) NW of Argentina (Oliva and Di Iorio 2008)
–	Antennal club entirely black	*Oxelytrum anticola* Gérin-Méneville Bolivia: La Paz; Oruro; Chile: Parinacota (Elgueta and Arriagada 1989); Ecuador: Latacunga; Quito. Perú: several localities (Peck and Anderson 1985)
5	Pronotum and elytra entirely black or dark brown. Apical antennomere yellow or orange. Paramera narrow, broadened at apical ¼, apices rounded, weakly turned inwards. Median lobe shorter than paramera, parallel-sided, very thick, weakly acuminate towards bifid apex (Fig. 7D)	*Oxelytrum lineatocolle* (Laporte) Patagonia (Oliva and Di Iorio 2008). Chile: several localities; associated with Valdivian rainforest, often with *Nothofagus* forest (Peck and Anderson 1985).
–	Pronotum with reddish margins, disk black at least on part (Fig. 8A). Color of antennal club varying	7
6	Humeral humps rounded. Pronotum with strongly raised longitudinal costs. Elytra with the second elytral cost clearly raised in its whole length	7
–	Humeral humps dentate (Fig. 8B). Pronotum with obsolete costae. Elytra with second costa obsolete on elytral disk	8
7	Antennal club with apical antennomere yellow or orange. Elytral apices emarginate, with sutural angles acuminate	*Oxelytrum emarginatum* (Portevin) Brasil: Minas Gerais, Rio de Janeiro, Sao Paulo (Peck and Anderson 1985)
–	Antennal club black. Elytral apices not emarginate, sutural angles rounded or weakly produced. Paramera narrow, broadened at apical ¼. Median lobe much shorter than paramera, parallel-sided, apex shaped as a straight angle (Fig. 10E)	*Oxelytrum erythrurum* (Blanchard) Argentina: Chaco-Pampean plain, reaching the mountainous areas in the NW through the valleys (Oliva and Di Iorio 2008). Bolivia: Beni; S. Cruz. Brasil: Amapá; Amazonas; M. Grosso; Minas Gerais; Pará; R. de Janeiro; Rondonia; Sao Paulo. Colombia: Amazonas; Cundinamarca; Norte de Santander. Ecuador: Manabí; Napo; Pastaza; Pichincha. French Guiana. Guyana. Perú: Loreto; Junín. Venezuela: Amazonas; Aragua; Zulia (Peck and Anderson 1985).
8	Pronotum with the postcoxal lobe yellowish. Pronotal disk darkened on the central portion only. Elytral apices produced, blunt. Paramera narrow, acuminate, weakly turned inwards. Median lobe somewhat shorter than paramera, spindle-shaped, apex acuminate (Fig. 9C)	*Oxelytrum cayennense* (Sturm) Argentina: Tucumán; Bolivia: Cochabamba: Chapare; Brasil: Minas Gerais; Rio de Janeiro; Sao Paulo (Peck & Anderson).
–	Pronotum with postcoxal lobe entirely black. Pronotal disk entirely black (Fig. 8A, 8B). Elytral apices produced, acuminate (Fig. 8C). Paramera very narrow, broadened and slightly turned inwards at apical ^1/8^. Median lobe very little shorter than paramera, thick, spindle-shaped, apex abruptly narrowed, bifid (Fig. 9A). Internal sac bearing a pair of sclerites (Fig. 9B)	*Oxelytrum discicolle* (Brullé) Argentina: Misiones to USA: Texas (Peck and Anderson 1985).

**Figure 5.**
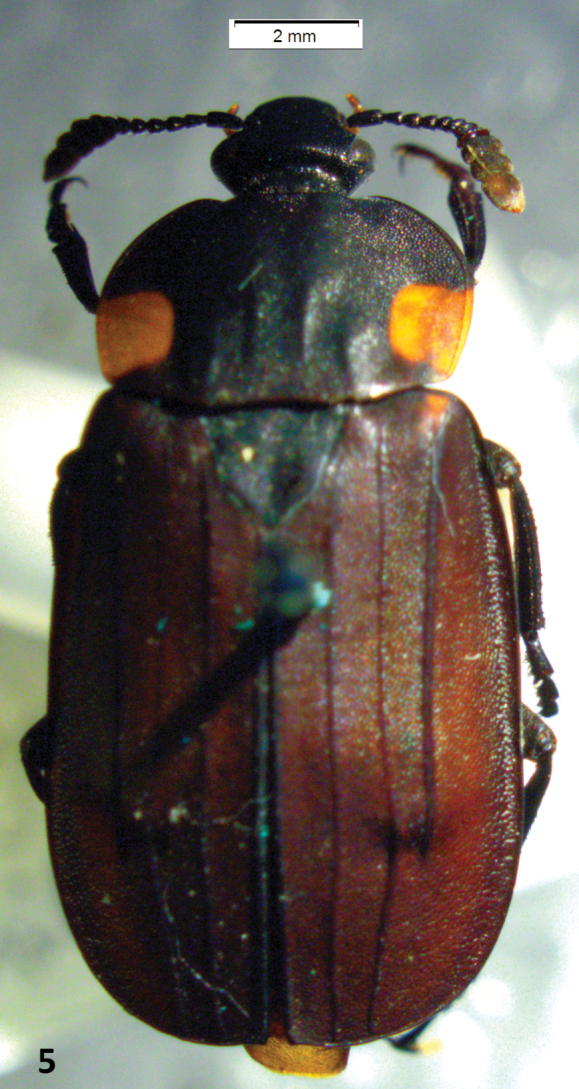
*Oxelytrum biguttatum*, dorsal aspect, showing pronotum with a pair of reddish spots and broadly rounded elytral apices. Scale bar = 2 mm.

**Figure 6.**
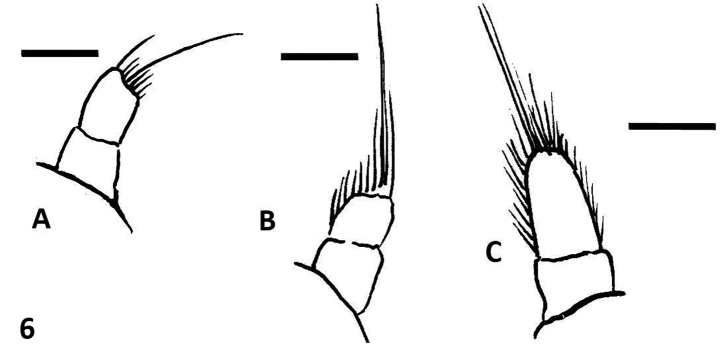
Female styli: line drawings. **A** *Oxelytrum selknan* **B** *Oxelytrum biguttatum* **C** O. lineatocolle. Scale bars= 0.2 mm.

**Figure 7.**
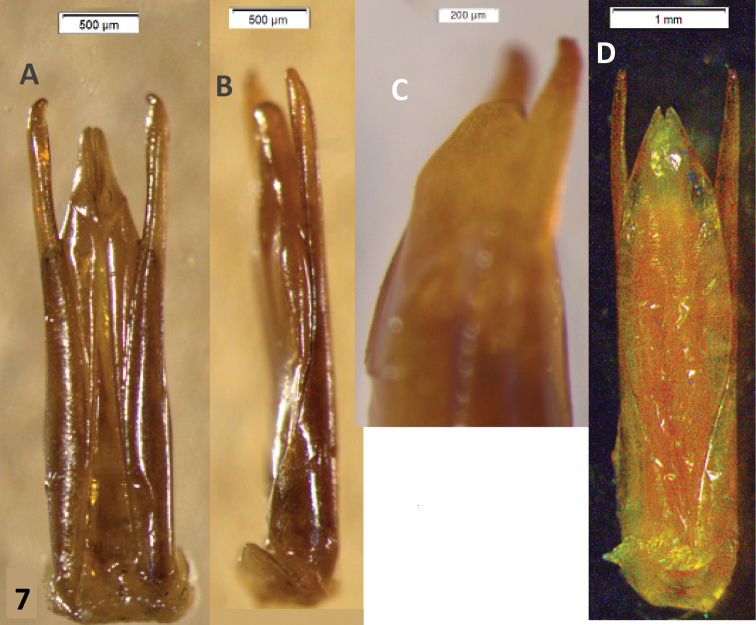
*Oxelytrum apicalis* and *Oxelytrum lineatocolle*, male genitalia. **A** *Oxelytrum apicalis*, male genitalia in dorsal aspect, showing median lobe shorter than paramera, acuminate to a narrowly rounded apex. Scale bar = 0,5 mm. **B** *Idem*, lateral aspect. Scale bar = 0, 5 mm. **C** *Idem*, apex of median lobe in lateral aspect, showing angular ventral ridge **D** *Oxelytrum lineatocolle*, male genitalia in dorsal aspect, showing broad median lone with bifid apex. Scale bar = 1 mm.

**Figure 8.**
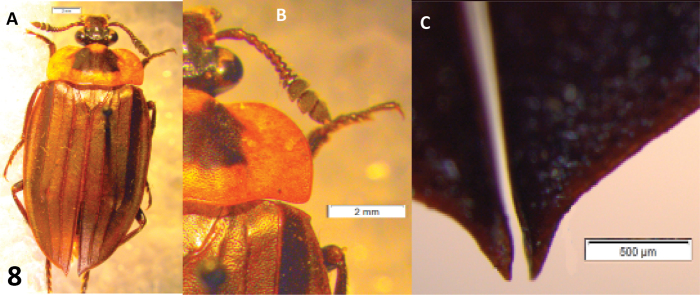
*Oxelytrum discicolle*, external morphology. **A** dorsal aspect, showing reddish pronotal disk, obsolete pronotal ridges and produced elytral apices. Scale bar = 2 mm. **B** right half of head and pronotum showing prominent compound eye and dentate humeral hump. Scale bar = 2 mm. **C** elytral apices, produced and acuminate. Scale bar = 0.5 mm.

**Figure 9.**
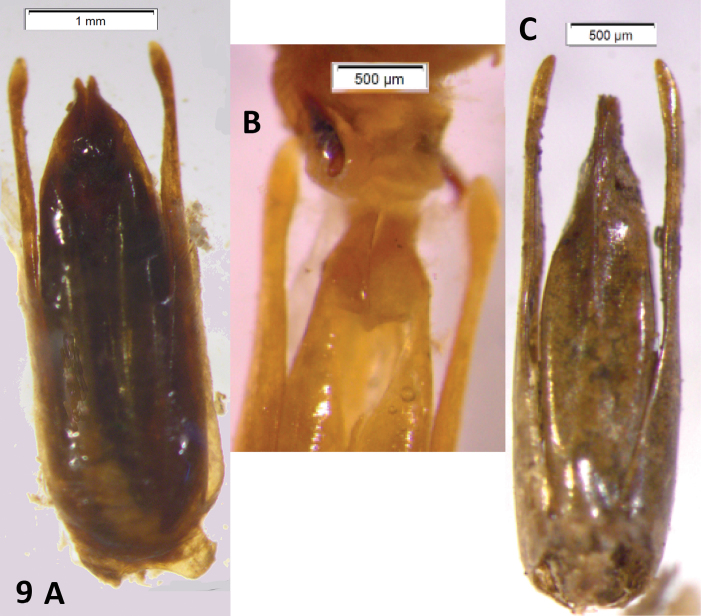
*Oxelytrum discicolle* and *Oxelytrum cayennense*, male genitalia. **A** *Oxelytrum discicolle*, male genitalia in dorsal aspect, showing narrow paramera dilated on apical one-eight and median lobe shorter than paramera, very broad, spindle-shaped. Scale bar = 1 mm. **B** Idem, apices of distal pieces with partly everted internal sac, showing a pair of sclerites. Scale bar = 0.5 mm. **C** *Oxelytrum cayennense*, male genitalia in dorsal aspect, showing paramera curved inwards, median lobe shorter than paramera, gradually acuminate. Scale bar = 500 μm.

**Figure 10.**
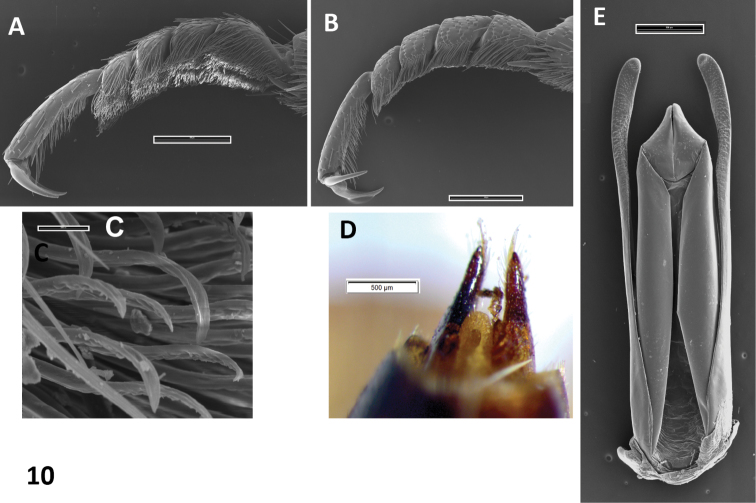
*Oxelytrum erythrurum*. **A** male protarsus, showing soles of hairs. SEM image. Scale bar = 500 μm. **B** female protarsus, without soles. SEM image. Scale bar = 500 μm. **C** detail of hairs. SEM image. Scale bar = 10 μm. **D** female styli. Scale bar= 0.5 mm. **E** male genitalia in dorsal aspect, showing paramera broadened on apical ¼, median lobe shorter than paramera, parallel-sided with triangular apex. SEM image. Scale bar = 500 μm.

## Discussion

*Oxelytrum selknan* appears to be a case of speciation through geographic isolation. The discovery of a new species which differs from a described one only by the male genitalia suggests that there may be more undescribed species. The internal sac is often poorly preserved in pinned specimens; work upon fresh material would probably allow a fuller description of all the species. Also, most specimens have poorly determined localities. An extensive sampling over the country would make clear the distribution of the species, giving a better idea of the usefulness of each as a forensic indicator.

## Supplementary Material

XML Treatment for Oxelytrum selknan

XML Treatment for Oxelytrum biguttatum
